# Prevalence of Type of Attachment and Morphological Variations of Median Maxillary Labial Frenum Among Children

**DOI:** 10.1155/ijod/8855769

**Published:** 2025-07-02

**Authors:** Rejina Shrestha, Amar Bhochhibhoya, Tekendra Chaulagain

**Affiliations:** ^1^Department of Dental Surgery, Kanti Children's Hospital, NAMS, Kathmandu, Nepal; ^2^Dental Department, Maharajgunj Medical Campus, TUTH, Kathmandu, Nepal

**Keywords:** classification, morphology, type of attachment

## Abstract

**Introduction:** Aberrant maxillary frenal attachment pose a problem of midline diastema and gingival recession. In children, they also cause difficulty in maintaining oral hygiene, accumulation of plaque which ultimately results in dental caries. Thus, a study was conducted with the objective of determining the prevalence of frenum in children.

**Method:** A total of 369 children from Kanti Children's Hospital (KCH) visiting the dental department were recruited in the study. The frenum was examined and classified by Placek's classification and Sewerin's classification. Chi-square test was used for showing association between frenum with age and gender.

**Result:** The most prevalent frenum type on the basis of type of attachment was gingival (51.2%), followed by the mucosal type (43.6%), papillary penetrating type (2.7%), and papillary type (2.5%). According to the morphological type, the most common frenum was found to be simple frenum (81%), simple frenum with appendix (7.9%), frenum with nodule (6.5%), persistent tectolabial (2.2%), simple with nichum (1.6%), and bifid labial frenum (0.8%).

**Conclusion:** The most common frenum was gingival frenum and simple frenum in children. Morphological frenum variants were associated with gender. The attachment of frenum fibres showed association with age. As the age progressed, the frenum was observed to migrate apically.

## 1. Introduction

Labial frenum is a fold of mucosa which attach lips and cheeks to alveolar process of the jaws [1]. The median maxillary labial frenum is located in the midline between the upper central incisors [1]. It is also called the superior labial frenum [2]. It consists of loose connective tissue and epithelium with or without muscle [2–4]. It embryologically originates as remnant of the central cells of the vestibular lamina at the midsagittal area [5]. It limits the movements of the upper lips and helps in its stability. It is a normal anatomical structure, and is found in different morphologic variations with different types of attachment.

According to Mirko et al. [6], based on the type of attachment, frenum are of four types: mucosal, gingival, papillary, and papilla penetrating. The papillary and papillary penetrating frenal attachments are considered to be pathological only after the mixed dentition period [7].

According to Sewerin [8], based on the morphological variations, frenum are of eight types: normal frenum, normal frenum with a nodule, normal frenum with appendix, normal frenum with nichum, bifid labial frenum, persistent tectolabial frenum, double frenum, and wider frenum. The variations in the frenum may be either familial or irregularity during development. These frena are not pathological conditions and do not require biopsy for diagnosis. To avoid misdiagnosis and unnecessary treatment, the dental practitioner must be familiar with different positions and morphologies of the frenum [1].

High frenal attachment is an etiological factor of midline diastema and gingival recession. During orthodontic treatments, high frena can complicate orthodontic spacing and also cause post orthodontic relapse [9–11].

In young children, the presence of wide and thick frenum is quite common. This restricts cleansability of the area which causes plaque accumulation ultimately leading to dental caries in the primary teeth. During growth, the frenum turns thin and wide. However, unlike ugly duckling stage, it is not self-correcting and requires surgical correction if it remains wide and thick [12]. Other indication for removal of maxillary frena in infants includes lip tie, which causes difficulty in breast feeding and bottle feeding and improper latching [2, 13].

There is paucity of data on the prevalence of median maxillary frenum in children in Nepal. The purpose of this study is to determine the prevalence of the type of median maxillary frenal attachments and morphological variation in children in Nepalese population.

## 2. Materials and Methods

A cross sectional study was done among a total of 369 subjects, recruited from Dental Department, Kanti Children's Hospital (KCH). Children with an age range of 1–14 years who visited Dental Department of KCH for routine dental treatment, were included in the study from convenience sampling. Ethical clearance was taken from the Institutional Review Committee (IRC) of KCH. The duration of the study was 6 months (February–July 2024). The minimum sample size was computed using the formula:

  $$\begin{array}{c} n=z^{2}p\left(1-p\right)/d^{2}=1.96^{2}\times 0.6\times \left(1-0.6\right)/0.05^{2}=369, \end{array}$$

where *n* = sample size, *z* = standard normal deviate of 1.96 for a confidence level set at 95%, *p* = the prevalence set at 60% [14], and *d* = the standard error 0.05.

The inclusion criteria was children who visited Dental Department of KCH for routine dental treatment with an age range of 1–14 years. The exclusion criteria were surgeries, trauma, congenital anomaly, and developmental anomaly in the premaxillary region; any completed or undergoing orthodontic treatment and known use of any medication or any syndrome that affect the gingiva. Written informed consent was taken from the attendant of the participants who wanted to participate in the study. Demographic variables such as, age and gender were recorded on the proforma. Under adequate light, the site of attachment of frenum with its morphology was examined by distending the upper lip upward with the index finger and thumb of both hands in a gentle manner. Mouth mirrors were used to view the palatal surface of the children for assessing any palatal attachment of the frenum.

The type of frenal attachment were categorized as mucosal, gingival, papillary, and papillary penetrating based on the classification by Mirko et al. [6] and simple, persistent tectolabial, frenum with nodule, frenum with appendix, double frenum, frenum with nichum, bifid frenum, and frenum with two or more variations by Kotian and Jeevanandan [15].

The recorded data were compiled and entered into Statistical Package for the Social Sciences-SPSS version 23.0 for statistical analysis. The data was analyzed using descriptive statistics (frequency and percentage). The obtained results were presented in the form of bar diagrams. The age group was divided into two age groups: Group 1 (1–7 years) and Group 2 (8–14 years). The chi-square test was performed to find association between the various categorical variables.

## 3. Results

**Figure 1:**
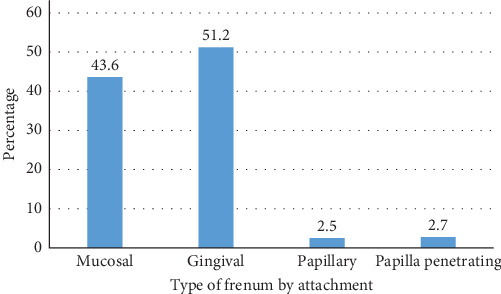
Prevalence of frenum on the basis of type of attachment.

**Figure 2:**
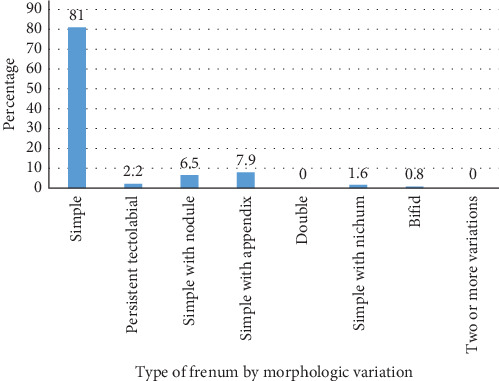
Prevalence of frenum on the basis of morphology variation.

A total of 369 children were included in the study. There were 223 (60.4%) male participants and 146 (39.6%) female participants. The average age was 6.6 years with an age range of 1–14 years. There were 236 (64%) children in the age range 1–7 years, and 133 children (36%) in the age range 8–14 years. The prevalence of type of attachment and morphological variations of median maxillary labial frenum among children are shown in Figures 1 and 2, respectively.

The results showed that the most common type of frenum was gingival type (51.2%) on the basis of attachment, and simple frenum (81%) on the basis of morphologic variation by Sewerin's classification. Double frenum and frenum with two or more variations were not reported in the study.

The distribution of types of frenum on the basis of Placek classification and Sewerin classification are shown in Tables 1 and 2, respectively. The most common type of frenum was gingival type and simple type in both the genders. A statistically significant value (*p* = 0.02) was observed in the frenum in the morphology of frenum on the basis of morphology.

The most common type of frenum was simple frenum in both the age groups on the basis of morphology. On the basis of attachment, gingival frenum had a higher preponderance in the age group 1–7 years, and mucosal frenum was more prevalent in the higher age group. A statistically significant value (*p* = 0.003) was observed in the frenum in the type of attachment of frenum on the basis of age.

## 4. Discussion

The study was conducted at KCH to assess the prevalence of frenum among children based on the type of attachment and the morphology. The first published report on the present topic was done by Bergese [16] in 1966, even before the proposal of classification by Mirko et al. [6] and Sewerin [8]. The frenum was classified as frenum in the attached gingiva (58.2%), attached to the alveolar mucosa (5.5%), the mucogingival junction (12.6%), the sulcus of the free gingiva (10.5%), the free gingiva (7.1%), and the palatal papilla (4.8%). Since then, several previous studies have been done on frenum among children which have shown varying results.

In our study, the most common frenum type was gingival (51.2%). This was followed by the mucosal type (43.6%), papillary penetrating type (2.7%), and papillary type (2.5%). The result is consistent with other studies done by Kramer et al. [17], Joshi et al. [14], Upadhyay and Ghimire [18], Kotian and Jeevanandan [15], and Pandiyan and Hedge [19]. However, in some studies done by Joshi et al. [14], Kotian and Jeevanandan [15], Jankzuk and Banach [20], the mucosal type was found to be more common followed by the gingival type. It is to be noted that the age group considered in the study done by Joshi et al. [14] was 6–16 years, and the age group in the study done by Janczuk and Banach [20] was 15–17 years. In contrast to the present study, papillary penetrating was found to be the least prevalent frenum in the studies done by Christabel [7], Divater et al. [21], and Upadhyay and Ghimire [18]. In a study done by Kaimenyi [22], frena attached on the papilla were not observed at all.

No association was established between frenum and gender in the present study which is similar to the result found in studies of Christabel [7], Upadhyay and Ghimire [18], Divater et al. [21], and Boutsi and Tatakis [27]. Gingival attachment was found in the younger age group and mucosal in the older category, which was statistically significant. Similar results have been observed in other studies [7, 21]. It can be perceived that there is a tendency of the frenum to migrate apically with age [18, 21]. This may be due to the increase in the alveolar ridge height during primary dentition, and vertical growth acceleration during the eruption of the permanent maxillary central incisors [18]. Due to this change in the frenum type with age, a delay in surgical interventions in the early ages of life has been suggested to avoid overtreatment [17]. A systematic review done by Tadros et al. [23] in 2022, explained that corrections of frenum should not be done until permanent lateral incisors have erupted, as there are possibilities of spontaneous closure of the midline diastema. The current literature also recommends to delay frenectomy until the orthodontic treatment has been completed to prevent diastema relapse.

According to the morphological type, the most common frenum was found to be simple frenum (81%), followed by simple frenum with appendix (7.9%), frenum with nodule (6.5%), persistent tectolabial (2.2%), simple with nichum (1.6%), and bifid labial frenum (0.8%). Simple frenum was found to be the most common frenum in majority of the studies [15, 24]. The second most common frenum was persistent tectolabial in studies done by Diaz Pizan et al. [24], Birader et al. [25]; simple with appendix in study done by Kotian and Jeevanandan [15], and frenum with nodule in studies done by Joshi et al. [14] and Rathod et al. [26]. Statistical analysis showed a lack of association between type of morphological frenum and age. The variations were found to be significantly different among the gender. This result is in accordance with other studies by Kramer et al. [17].

The limitations of the study are the small sample size, convenience sampling method, and lack of assessment of different factors such as social factors. The variations in percentage may be due to diverse population characteristics and different variations in the same population owing to different race, caste, and ethnicity. Variation also occurs according to age criteria defined as there is heterogeneity in the included population. Some studies have included only primary dentition, some have included mixed and permanent dentition, while some have included only adolescent population. Also morphology of inverted *Y* type, trifid frenum were neither reported nor included in the study.

## 5. Conclusions

The most common frenum in children is simple type and gingival type. The dental practitioner should be well informed about the different types and attachment of frenum to avoid inadvertent treatment. Frena are an important part of the oral cavity and an etiology of gingival recession and midline diastema. They should be checked during routine oral examination unnecessary biopsies should not be done.

## Figures and Tables

**Table 1:** Distribution of types of frenum based on gender.

Types of frenum	Gender		Total, *n* (%)	*p*-Value
	Male, *n* (%)	Female, *n* (%)		
Placek classification				
Mucosal	101 (7.4)	60 (16.3)	161 (43.6)	0.61
Gingival	113 (30.6)	76 (20.6)	181 (59.2)	
Papillary	4 (1.1)	5 (1.3)	9 (2.5)	
Papilla penetrating	5 (1.4)	5 (1.3)	10 (2.7)	
Sewerin classification				
Simple	171 (46.3)	128 (34.7)	299 (81)	0.02^*∗*^
Persistent tectolabial	5 (1.4)	3 (0.8)	8 (2.2)	
Simple with nodule	16 (4.3)	8 (2.2)	24 (6.5)	
Simple with appendix	26 (7.1)	3 (0.8)	29 (7.9)	
Double	0 (0)	0 (0)	0 (0)	
Simple with nichum	4 (1.1)	2 (0.5)	6 (1.6)	
Bifid	1 (0.3)	2 (0.5)	3 (0.8)	
Frenum with two or more variations	0 (0)	0 (0)	0 (0)	

*⁣* ^*∗*^ *p*-Value < 0.05 which shows statistical significance.

**Table 2:** Distribution of types of frenum based on age.

Types of frenum	Age		Total, *n* (%)	*p*-Value
	1–7 years, *n* (%)	8–14 years, *n* (%)		
Placek classification				
Mucosal	87 (23.6)	74 (20)	161(43.6)	0.003^*∗*^
Gingival	131 (35.5)	58 (15.7)	181 (59.2)	
Papillary	8 (2.2)	1 (0.3)	9 (2.5)	
Papilla penetrating	10 (2.7)	(0)	10 (2.7)	
Sewerin classification				
Simple	190 (51.5)	109 (29.5)	299 (81)	0.202
Persistent tectolabial	7 (1.9)	1 (0.3)	8 (2.2)	
Simple with nodule	11 (3)	13 (3.5)	24 (6.5)	
Simple with appendix	21 (5.7)	8 (2.1)	29 (7.9)	
Double	0 (0)	0 (0)	0 (0)	
Simple with nichum	5 (1.4)	1 (0.3)	6 (1.6)	
Bifid	2 (0.5)	1 (0.3)	3 (0.8)	
Frenum with two or more variations	0 (0)	0 (0)	0 (0)	

*⁣* ^*∗*^ *p*-Value < 0.05 which shows statistical significance.

## Data Availability

The data that support the findings of this study are available from the corresponding author upon reasonable request.
